# Semantic and Generalized Entropy Loss Functions for Semi-Supervised Deep Learning

**DOI:** 10.3390/e22030334

**Published:** 2020-03-14

**Authors:** Krzysztof Gajowniczek, Yitao Liang, Tal Friedman, Tomasz Ząbkowski, Guy Van den Broeck

**Affiliations:** 1 Department of Artificial Intelligence, Institute of Information Technology, Warsaw University of Life Sciences-SGGW, 02-776 Warsaw, Poland; tomasz_zabkowski@sggw.pl; 2 Computer Science Department, University of California, Los Angeles, CA 90095, USA; yliang@cs.ucla.edu (Y.L.); tal@cs.ucla.edu (T.F.); guyvdb@cs.ucla.edu (G.V.d.B.)

**Keywords:** deep learning, semantic loss, generalized entropy loss, machine learning

## Abstract

The increasing size of modern datasets combined with the difficulty of obtaining real label information (e.g., class) has made semi-supervised learning a problem of considerable practical importance in modern data analysis. Semi-supervised learning is supervised learning with additional information on the distribution of the examples or, simultaneously, an extension of unsupervised learning guided by some constraints. In this article we present a methodology that bridges between artificial neural network output vectors and logical constraints. In order to do this, we present a semantic loss function and a generalized entropy loss function (Rényi entropy) that capture how close the neural network is to satisfying the constraints on its output. Our methods are intended to be generally applicable and compatible with any feedforward neural network. Therefore, the semantic loss and generalized entropy loss are simply a regularization term that can be directly plugged into an existing loss function. We evaluate our methodology over an artificially simulated dataset and two commonly used benchmark datasets which are MNIST and Fashion-MNIST to assess the relation between the analyzed loss functions and the influence of the various input and tuning parameters on the classification accuracy. The experimental evaluation shows that both losses effectively guide the learner to achieve (near-) state-of-the-art results on semi-supervised multiclass classification.

## 1. Introduction

On the one hand, supervised learning uses labeled (marked) data to train a model that gives accurate forecasts of data that the model has never seen before, e.g., classification and regression [1,2]. On the other hand, unsupervised learning takes unlabeled data as an input and prepares a model based on the patterns or based on the dataset structure, e.g., dimensionality reduction, detecting outliers, and clustering [3,4]. Semi-supervised learning is halfway between unsupervised learning and supervised learning, i.e., there are both labeled and unlabeled data. Usually, it is assumed that unlabeled data constitute the majority of the dataset [5]. Semi-supervised learning is assumed to be supervised learning with additional information on the distribution of examples. Alternatively, it can be also be an extension of unsupervised learning guided by some limitations or constraints [6,7].

Deep learning has attracted considerable attention in recent years [8], a relatively broad class of machine learning (ML) techniques use (complex) artificial neural architectures for classification [9]. Such approaches encode nonlinear information through several hierarchical layers, thus, assimilating problems at different levels of abstraction. In practice, one is more likely than not to face the curse of “overfitting”. This problem is usually solved using regularization which is the process of entering additional information to manage this inevitable gap between a training error and a test error [10]. Regularization is often carried out by augmenting the loss function (e.g., mean square error or cross-entropy error) by a so-called regularization term, which prevents the model from over-optimizing the loss function estimated at a finite set of sampling observations. From a statistical point of view, regularization is interpreted as a prior distribution that reflects our expert knowledge or belief regarding a model. For example, this knowledge can take the form of a constraint (or sentence) in Boolean logic. It can be as simple as an exactly-one constraint for one-hot output encodings, which is the way of converting categorical data to numerical data in multiclass classification problems [11].

This constraint is ubiquitous in the multiclass classification tasks. This means that for a given example exactly one binary class/label must be true. The ML community has made great progress in this task by inventing various representations and their associated regularization terms [12]. In order to maintain this progress and reduce the need for more labeled data, there is growing interest in using unlabeled data to increase the predictive power of classifiers by incorporating a semantic loss function for this task [6,7]. The semantic loss defined in this setting with respect to the exactly-one constraint obtains a learning signal from a huge amount of unmarked data. The main idea is that the semantic loss helps to improve classification of the unlabeled data. Therefore, the first main goal of this article is to verify whether this simple addition to the loss function of standard deep learning architectures provides significant improvements over if this new regularization term is not added (i.e., unlabeled data is not utilized).

In the machine learning context, information and entropy are useful tools that serve as the basis for a number of applications including selecting features, building decision trees, training artificial neural networks and, more generally, fitting classification models [13]. Apart from the most commonly used entropy in this context which is the Shannon’s entropy [14], one can distinguish the Rényi entropy [15]. The definition of the Rényi entropy consist of a Q parameter (also called the generalization parameter) which for special cases generalizes the Shannon’s entropy, the Hartley entropy, the collision entropy, and the minimum entropy [16]. The Rényi entropy has found interesting applications [17,18] including the parametric weighting of the probabilities that endows data analysis with additional flexibility. In this context, the second main goal of this article is to examine, in the same spirit as the first question, whether the addition of the generalized entropy loss function to the loss function provides significant improvements over if this generalized regularization term is not added (i.e., unlabeled data is not utilized).

To these two ends, we evaluate our proposed methods over an artificially created dataset and two commonly used benchmark datasets (i.e., MNIST [19] and Fashion-MNIST [20]) with the expectation that the following furthermore research questions can also be addressed:

- If the two analyzed regularization terms prove to be effective in semi-supervised classification tasks, which loss function provides the best results?
- What is the relation between semantic loss function and generalized entropy loss function?
- What is the impact of the input and tuning parameter values on both proposed approaches on the final results?

In summary, the goal of this article is to assess the performance of the generalized entropy and semantic losses, and to highlight their effects, not to achieve a state-of-the-art performance in relation to a specific problem. In order to do this, the adopted neural network architecture, in addition to the loss term, must set up the baseline points (please see Section 4.3 and Section 4.5) to the performance of a semi-supervised method. In other words, to have a principled comparison, the adopted neural network architecture shall be identical with the recent state-of-the-art baseline.

The remainder of this paper is organized as follows: Section 2 and Section 3 provide an overview of the similar research problems and the theoretical frameworks of the semi-supervised learning, the artificial neural networks, and the two loss functions used in this article; in Section 4, the research framework is outlined, including the details of numerical implementation, dataset characteristics, and model performance measures; Section 5 outlines the experiments and presents the discussion of the results; and the paper ends with concluding remarks in Section 6.

## 2. Preliminaries

### 2.1. Semi-Supervised Learning

In many situations, marked data is missing. Labels are difficult to obtain because they require human annotators, sophisticated devices, or expensive and lengthy experiments, and therefore semi-supervised learning is very useful. In particular, its application includes the following problems [3,5,21]: speech recognition, natural language parsing, spam filtering, video surveillance, and image categorization. The algorithms are divided into the following categories [3,5,21]: self-training, generative models, co-training, graph-based algorithms, and multi-view learning. In general, those algorithms assume the following data properties:

- Manifold assumption, the data lie approximately on a manifold of much lower dimension than the input space. This assumption allows the use of distances and densities which are defined on a manifold;
- Continuity assumption, the algorithm assumes that (after transformed to a lower dimension) the points which are closer to each other are more likely to have the same output label;
- Cluster assumption, (after transformed to a lower dimension) the data is divided into discrete clusters and points in the same cluster are more likely to share an output label.

In traditional supervised learning tasks, we are presented with an ordered set of l marked observations $D_{L}={\left\{\left(x_{i},y_{i}\right)\right\}}_{i=1}^{l}$. Each observation $\left(x_{i},y_{i}\right)$ consists of an object $x_{i}\in X^{p}$ from a given p-dimensional input space $X^{p}$ and has an associated label $y_{i}$, where $y_{i}$ is a real value for the regression or (as in this article) a category for the classification task, i.e., $y_{i}\in \left\{1,\ldots ,k\right\}$. On the basis of a set of these observations, usually referred to as training data, supervised learning methods try to deduce a function that can successfully determine the label $y^{*}$ of some previously invisible input $x^{*}$. However, in many real classification tasks we also have access to a set of u observations, $D_{U}={\left\{\left(x_{i}\right)\right\}}_{i=l+1}^{l+u}$, whose labels are unknown.

**Figure 1:**
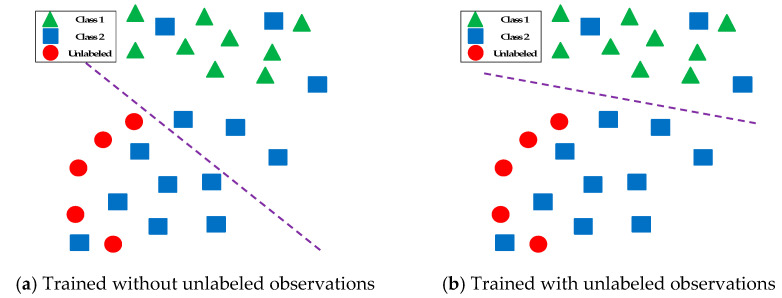
A toy example of binary classification in the presence of unlabeled data.

Figure 1 provides further details on the use of unlabeled data for classification of an artificial problem with two classes. Any supervised learning algorithm is likely to obtain a line presented on the left-hand side of the figure as the decision boundary. However, this is far from the optimal decision boundary. As presented on the right-hand side of this figure, the clusters that we infer from unlabeled observations help significantly to determine the decision boundary.

The primary objective of semi-supervised learning is to use unlabeled observations to develop better learning procedures. However, this is not always easy or even possible [22]. As mentioned earlier, unmarked observations are useful only if they contain relevant information for predicting labels that are not included in the labeled data itself or cannot be easily extracted. To apply any semi-supervised learning method in practice, the algorithm must be able to extract such information.

### 2.2. Deep Neural Networks

Deep learning is a subfield of machine learning that are concerned with algorithms inspired by the structure and function of the brain, called artificial neural networks, along with representation learning. Deep neural networks (DNNs) such as deep multi-layer perceptrons (MLPs), deep belief networks (DBNs), long short-term memory neural networks (LSTMs), recurrent neural networks (RNNs), and convolutional neural networks (CNNs) [9,23,24] have been applied to a variety of fields including computer vision, speech recognition, natural language processing, audio recognition, social network filtering, machine translation, bioinformatics, drug design, medical image analysis, material inspection, and board game programs, where they have produced results comparable to, and in some cases surpassing, human expert performance [25,26].

**Figure 2:**
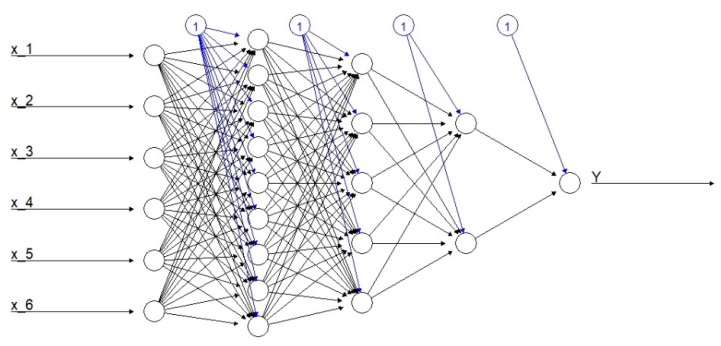
An exemplary feedforward deep multi-layer perceptron.

Importantly, multiple deep learning architectures exist and, as interest and research in this area increases, the field will continue to flourish. However, fundamental to of all these methods is the feedforward multi-layer perceptron (MLP). Feedforward MLPs consist of densely connected layers, in which the input affects each subsequent layer up until the final output layer. Figure 2 presents an example of MLP with six input neurons (features), one output neuron (target), and three hidden layers consisting of nine, five, and two hidden neurons, respectively (with additional biases marked in blue). There is no well-defined approach to choose the number of hidden layers and nodes, and hence they effectively are the first of many hyper-parameters to tune. The choice of output layer is driven by the modeling task. For example, for a binary classification task the output layer contains only one node predicting the probability of success, while for a multiclass classification task the output layer consists of the same number of nodes as the number of classes being predicted.

A key element of a DNN is the activation process. In the human brain, a biological neuron receives inputs from many adjacent neurons and when these inputs exceed a certain threshold, the neuron is activated, which suggests there is a signal. The activation function is simply a mathematical function that determines whether there is enough information in a node to raise a signal to the next layer. There are many activation functions in DNN to choose from, for example, identity, sigmoid, softmax (please refer to Section 4.2), but, currently, the most popular is rectified linear unit (ReLU) [27]:

(1) $$f\left(x\right)=\{\begin{array}{c} 0,\;\;x<0\\ x,\;\;x\geq 0 \end{array},$$

especially for rectangular data, such as for image classification.

During training a DNN selects a batch of observations, randomly assigns weights to all node connections and predicts the results. The backpropagation process of the neural network is in place to assess its own accuracy and to adjust automatically the weights for all node connections to improve that accuracy. This process itself requires two things. First, one must establish a loss function L to measure performance, i.e., this might be the mean square error (MSE) or cross entropy (please refer to Section 3.2) [1]. Secondly, on each forward pass, the DNN measures its performance based on the selected loss function. Then, the DNN works backwards through layers, calculates the gradients of the loss in relation to the network weights, adjusts the weights slightly in the opposite direction to the gradients, takes the next batch of observations to go through the model, flushes and repeats until the loss function is (locally) minimized [13]. This process is also known as mini-batch stochastic gradient descent with representatives such as adaptive gradient algorithm (AdaGrad), root mean square propagation (RMSProp), or adaptive moment estimation (Adam) [28].

It should be noted that DNNs require that all feature inputs are numerical, i.e., they have to be numerically encoded using, for example, one-hot encoded (target variable in our case) or integer label encoded. Due to the data transformation process performed by DNN, they are very sensitive to the individual scale of function values. Therefore, one should use normalized features in advance e.g., by standardization (i.e., zero mean and unit variance) or range normalization (i.e., all features are transformed to between [0,1]). Unfortunately, the scaling problem also arises in the intermediate layers, because the distribution of activations is constantly changing during the training. This slows down the training process, as each layer has to learn to adapt to the new distribution at each stage of the training. This problem is formally known as the internal shift of the covariable. Fortunately, in order to overcome this problem one can use batch normalization which is a method that normalizes the inputs of each layer [29].

DNNs can include local or global pooling layers to streamline the underlying computation. Pooling layers reduce data dimensions by combining the results of neuron clusters at one layer into a single neuron in the next layer. For example, max pooling uses the maximum value from each of a cluster of neurons at the prior layer.

Finally, placing constraints on a model’s complexity (as a regularization) is a common way to mitigate overfitting [13]. There are two common approaches, both of them are applicable in DNNs in a similar manner to other methods such as Ridge or Lasso regression. One can use the L_1_ or L_2_ penalty to add costs proportional to the size of the node weights. Regularizing the weights forces small signals (noise) to have weights almost equal to zero and allows only consistently strong signals to have relatively higher weights. More specifically, for some hyper-parameter w, the new overall loss becomes:

(2) Loss function=existing loss +w∗regularization term.

In addition to the abovementioned methods, one can distinguish other commonly used regularization approaches such as dropout, data augmentation, or early stopping.

### 2.3. Propositional Logic

In order to formally define semantic loss (Section 3.1), first, the concept of propositional logic should be introduced. Let upper case letters (X,Y) denote Boolean variables and lowercase letters (x,y) denote their realizations (X=0 or X=1). Bold uppercase letters (X,Y) denote the sets of variables, and bold lowercase letters (x,y) denote their joint realizations. A variable (x) or its negation (¬x) is a literal. A logical sentence (α or β) is constructed in the usual way, from variables and logical connectives (∧, ∨, etc.), and is also called a formula or constraint [6]. A state or world x is an instantiation to all variables X. A state x satisfies a sentence α, denoted x⊨α, if the sentence evaluates to be true in that world, as defined in the usual way [7]. A sentence α entails another sentence β, denoted α⊨β if all worlds that satisfy α also satisfy β. A sentence α is logically equivalent to sentence β, denoted α≡β, if both α⊨β and β⊨α [6,7].

## 3. Theoretical Framework of the Semantic and the Generalized Entropy Loss Functions

### 3.1. Semantic Loss Function

The purpose of the semantic loss function is to fill in the gap between the continuous world of feedforward DNNs and the symbolic world of propositional logic. The semantic loss $L_{s}\left(\alpha ,p\right)$ is a function of the sentence α in the sentence logic, defined by the variables $Y=\left\{Y_{1},\ldots ,Y_{j},\ldots ,Y_{k}\right\}$ and probability vector p for the same variables Y [7]. Element $p_{j}$ is the predicted probability of the $Y_{j}$ variable and corresponds to one node in the output layer of the neural network. For example, the semantic loss between exactly one constraint α and the output vector p of the neural network shows how close the prediction p has exactly one output set to true (1) and all false (0), regardless of which output is correct [6].

In general, the semantic loss $L_{s}\left(\alpha ,p\right)$ should be proportional to the negative logarithmic probability of satisfying the constraint α when sampling the values of the variables in α according to p:

(3) $$L_{s}\left(\alpha ,p\right)\propto -log\underset{y⊨\alpha }{\sum }\underset{j:y⊨Y_{j}}{\prod }p_{j}\underset{j:y⊨\neg Y_{j}}{\prod }\left(1-p_{j}\right),$$

where y⊨α means that the assignment of y to the Y variables meets the sentence α, and $y⊨Y_{j}$ means that Y is set to true in the world y. In other words, this is the self-information about obtaining an assignment that meets the constraint [7].

When the constraint over the output space is simple (for example, there is a small number of solutions y⊨α), the semantic loss can be directly computed from Equation (4). Concretely, for the exactly-one constraint used in k-class classification, the semantic loss reduces to:

(4) $$L_{s}\left(exactly-one,p\right)\propto -log\overset{k}{\underset{j=1}{\sum }}p_{j}\overset{k}{\underset{m=1,m\neq j}{\prod }}\left(1-p_{m}\right),$$

where the value $p_{j}$ denotes the probability of class j as predicted by the neural network. The semantic loss for the exactly-one constraint is efficient and imposes no noticeable computation overhead in this study [7]. In general, for any given semantic loss, complex or simple, to achieve efficient computation, one can first compile its constraint α into a certain class of logical circuits [30], and then the time spent on computing the semantic loss is only linear in terms of the size of the circuit.

### 3.2. Generalized Entropy Loss function

Entropy is a well-known term in thermodynamics, statistical mechanics, and information theory. Although the concepts of entropy have deep interconnections, it took many years to develop the theory of statistical mechanics and information theory to make this connection visible. This article deals with information entropy, the theoretical formulation of information entropy. Entropy of information is sometimes called Shannon entropy in honor of Claude E. Shannon, who formulated many key ideas of information theory [14].

Entropy is a measure of unpredictability of the state, or equivalently, of its average information content. The intuition behind the quantification of information consists in measuring the amount of surprise in a given event. Those events that are rare (i.e., with low probability) are more surprising, and therefore have more information than those events that are common (i.e., with high probability). Rare events are more uncertain or more surprising and require more information to represent them than common events. In general, the concept of information entropy is defined as:

(5) $$H_{S}\left(p\right)=-\overset{k}{\underset{j=1}{\sum }}p_{j}\log p_{j},$$

where the above symbols are the same as for the semantic loss function. The value of the entropy depends on the following two parameters: (1) disorder (i.e., uncertainty), which is maximum when the probability $p_{j}$ for every $Y_{j}$ is equal and (2) the value of k. Shannon entropy assumes a tradeoff between contributions from the main mass of the distribution and the tail. To control both parameters, a generalization was proposed by Rényi [15] with the goal to provide a foundation for nonextensive statistical mechanics:

(6) $$H_{R_{Q}}\left(p\right)=\frac{1}{1-Q}\log\left(\overset{k}{\underset{j=1}{\sum }}p_{j}^{Q}\right).$$

With Shannon entropy, events with high or low probability have equal weights in the entropy computation. However, using Rényi entropy, for Q>1, events with high probability contribute more than low probabilities for the entropy value. Therefore, the higher the value of Q, the higher the contribution of high probability events is in the final result.

Each value of Q gives a possible entropy measure. All are additive for independent random variables and, for each discrete random variable, $H_{R_{Q}}$ is a monotone nondecreasing function of Q. Assuming that all the probabilities $p_{j}$ are positive, then, $H_{R_{0}}$ is known as the maximum entropy or Hartley entropy [31]. When Q=1 we get the more familiar Shannon entropy (i.e., in this limit the Shannon entropy is computed using Equation (5)). When the order Q is not specified, the default value is 2. This case is also called collision entropy and is used in quantum information theory [32]. Finally, in the limit as Q goes to ∞, the Rényi entropy converges to the negative log of the probability of the most probable outcome, i.e., minimum entropy.

### 3.3. Relation between Generalized Entropy and Semantic Loss Functions

**Figure 3:**
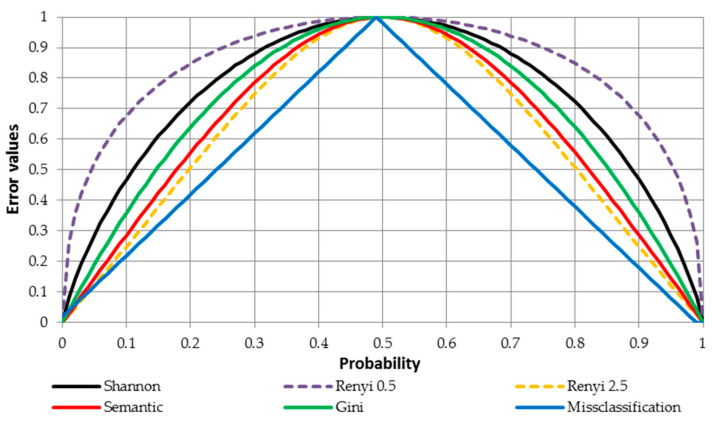
Relation between various classification errors for binary classification problem.

With regards to the binary classification task, there are three commonly used loss functions in machine learning algorithms, i.e., (1) Shannon entropy (Equation (5)), (2) Gini index of the form $\mathrm{Gini}\left(p\right)=1-\sum_{j=1}^{2}p_{j}$, and (3) the miss/classification error of the form $\mathrm{MissClass}\left(p\right)=1-\max\left(p_{1},p_{2}\right)$. The entropy is 0 if one class has a probability of 0, while the other class is 1, and the entropy is maximal for uniform class distribution. Similar to entropy, the Gini index is maximal if the classes are perfectly mixed. In practice, both losses yield very similar results. Furthermore, the miss/classification error is less sensitive to changes in the class probabilities (see Figure 3).

In order to present the relation between various losses/errors for binary classification problem, Figure 3 is prepared. In this figure the horizontal axis presents the probability that for a particular observation the true value equals 1 (while having two classes 0 and 1). The vertical axis presents values of a particular error/loss function. For instance, in these settings the Shannon entropy can be calculated as follows (this approach holds for other errors as well). Let’s assume that the probability for the class 1 equals 0.7. Then, the probability for the class 0 equals 1–0.7. Finally, error value equals $H_{S}=-\left(0.3*\log0.3+0.7*\log0.7\right)\approx 0.88$. The blue solid line presents misclassification error, the green solid line denotes Gini error, and the black solid line represents standard entropy loss (Shannon, Equation (5)). The semantic loss is depicted by the red solid line while generalized entropy (Rényi) loss is presented by the yellow and purple dashed lines for Q equaling 0.5 and 2.5, respectively. It should be noted that the line for Rényi loss for Q=1 would be the same as for Shannon entropy. The analysis of Figure 3 reveals, on the one hand, that the semantic loss is less sensitive to the class distribution than the standard Shannon entropy and the Gini index but, on the other hand, is more sensitive than the miss/classification error. It is important to note that as the Q parameter for Rényi loss increases, its sensitivity to the class distribution decreases (from yellow to purple line).

**Figure 4:**
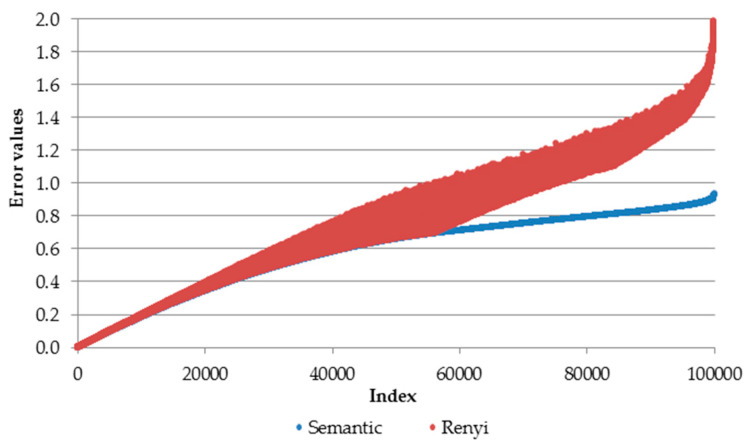
Relation between semantic and Rényi losses for a 10-class classification problem.

To obtain deeper insights into the relation between the semantic loss and the Rényi loss, we simulate an artificial dataset with the probabilities for a 10-class classification problem. A similar classification problem is analyzed in Section 5 where commonly used benchmark datasets with 10 classes are used. Probabilities are simulated from uniform distribution (*runif* function in R [33]) and they sum to 1. The final table consists of 100,000 rows (observations) and 10 columns (each for one class). Then, for this table we calclate semantic loss (Equation (4)) and Rényi loss (Equation (6)). For Rényi loss, we set the Q-parameter at 2, since according to Figure 3, for a two-class classification problem those two curves (points to be precise) would have almost the same relationship to the class probability distribution. The graphical relation between these two losses is presented in Figure 4.

The horizontal axis presents the index of a particular observation while the vertical axis depicts the Rényi and semantic loss values. Finally, the table with loss values is sorted in ascending order using semantic loss (blue). This results in increasing curves for both loss functions. Error values for both loss functions are relatively similar when both losses have relatively small values (up to approximately 0.5). Then, after this point both curves diverge from each other. Interestingly, the Rényi loss (red dotes) is bounded by the semantic loss, i.e., generalized entropy loss has values not less than semantic loss.

**Figure 5:**
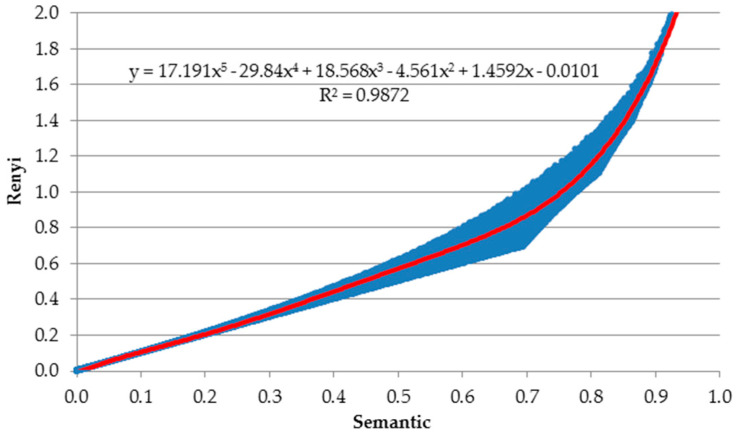
Fitted polynomial regression for semantic and Rényi losses.

Next, Figure 5 presents the relation between both losses, but right now having values of these errors on other axis. 

To quantify this relation, we estimate the polynomial regression using *lm* and *poly* functions in R (red solid curve). After testing and fitting different degree polynomial regressions (we try order of the polynomial from 1 up to 6 and we record $R^{2}$ of each model) we select the one having the greatest $R^{2}$ determination coefficient (to be precise if we change the degree from 5 to 6 there is no improvement in accuracy). The ultimate model has the following formula (see also Figure 5):

(7) $$R\text{é}\mathrm{nyi}=17.191{*\mathrm{semantic}}^{5}-29.84*{\mathrm{semantic}}^{4}+18.568*{\mathrm{semantic}}^{3}-4.561*{\mathrm{semantic}}^{2}\;+1.4592*{\mathrm{semantic}}^{2}-0.0101.$$

All estimated parameters are statistically significant at α=0.05. Other liner models’ assumptions are met as well. In addition to uniform distribution, we also test normal distribution and the conclusions remain the same. Moreover, we run this simulation for number of classes equal to 1, 3, and 5, and since the conclusions are the same, we stay on this point.

## 4. Research Framework and Settings

### 4.1. Datasets Characteristics

In this article, we use two benchmark datasets requiring similar data preparation. Since the two datasets are very similar, we are able to use the same structure for the deep network. The first dataset is the Modified National Institute of Standards and Technology (MNIST) digit dataset [19]. The MNIST dataset is divided into the following two subsets: The training dataset has 60,000 examples and the test dataset has 10,000 examples. All examples are small square 28 × 28 pixel (values from 0 to 255) grayscale images of handwritten single digits between 0 and 9.

The second dataset is Fashion-MNIST containing Zalando’s article images [20] which consists of a training set of 60,000 examples and a test set of 10,000 examples. Each example is a 28×28 pixels grayscale image associated with a label from 10 classes. It is intended to serve as a direct drop-in replacement of the original MNIST dataset, as it shares the same image size and structure of training and testing splits. Training and test examples are assigned to one of the following labels: 0-T-shirt/top, 1-Trouser; 2-Pullover, 3-Dress; 4-Coat, 5-Sandal, 6-Shirt, 7-Sneaker, 8-Bag and 9-Ankle boot.

### 4.2. Performance Measure

A proper evaluation is crucial for models built with any statistical learning algorithm. When designing a model to perform a multiclass classification task, we want the model to choose only one answer, e.g., the digit “8”. At the end of a deep network classifier, we get a vector of “raw output values”, for example, x=[−0.8, 1.2,−0.1] if a particular network has three outputs corresponding to each of the classes. However, we usually would like to convert these raw values into an understandable format, i.e., probabilities. In order to derive the probability of each class, $p_{j}$, the softmax function of the form is applied:

(8) $$p_{j}=\mathrm{softmax}\left(x_{j}\right)=\frac{e^{x_{j}}}{\sum_{j=1}^{k}e^{x_{j}}},$$

producing inter-related outputs which are always in the range [0,1] and add up to 1, and hence they form a probability distribution. This means, if we are using a softmax, in order for the probability of one class to increase, the probabilities of at least one of the other classes has to decrease by an equivalent amount. In order to assign the final class label for a given observation, a simple assumption is taken into account, i.e., the higher the probability the more likely the outcome:

(9) $$\hat{Class}=\arg\;\underset{j}{\max}\;p_{j},$$

where $p_{j}$ denotes the probability of class j being predicted by the deep network.

Eventually, to calculate the performance of any kind of predicting model for a multiclass classification problem, the following confusion matrix of k×k dimension is prepared:

According to Table 1, the accuracy measure can be computed, which is the proportion of the total number of predictions that are correct:

(10) $$\mathrm{Accuracy}=\frac{\sum_{j=1}^{k}{\mathrm{True}}_{j}}{\sum_{j=1}^{k}{\#}_{j}},$$

where ${\mathrm{True}}_{j}$ denotes the number of correctly classified instances belonging to the class j, and ${\#}_{j}$ stands for the number of instances in class j.

### 4.3. Numerical Implementation

All the numerical experiments presented below are prepared using Python programming language and TensorFlow [34] which is an end-to-end open source platform for machine learning. For comparison, we add Rényi and semantic losses to the same base models used in ladder nets [35], which currently achieve the state-of-the-art results on semi-supervised MNIST. The base model for both datasets is a fully-connected multilayer perceptron (MLP), with layers of size 784-1000-500-250-250-250-10 respectively. After every three layers, features are subject to a 2-by-2 maxpool layer with strides of 2. Furthermore, we use rectified linear unit (ReLu) as an activation function [27], batch normalization to improve the speed, performance, and stability of the networks [29], and Adam optimization algorithm that has been designed specifically for training deep neural networks [28] with a learning rate of 0.002. Because MNIST and Fashion-MNIST share the same image size and structure, methods developed in MNIST should be able to directly perform on Fashion without heavy modifications [6,7].

For the purpose of parameter tuning, from both original training sets we separate an additional validation set containing randomly chosen 10,000 samples (i.e., training datasets have the remaining 50,000 observations). Finally, the estimates for the performance measures for the training, validation, and test sets are produced with 10-fold cross-validation [1]. All further results are presented as an average over 10-folds.

### 4.4. Tuning of the Parameters

Our motivation in assessing the performance of the generalized entropy and semantic losses is not to achieve the state-of-the-art performance in relation to a specific problem, but rather to highlight their effect. For this purpose, we evaluate our method taking into account the following: (1) the problem is difficult in terms of the output space where the model cannot be matched directly from the data, and (2) deliberately use simple DNNs for evaluation. To answer the question about the effect of tuning the investigated parameters in terms of the quality of predictions we perform a grid search checking various combinations of the following parameters:

- Q-value $\in \left\{1\times 10^{-6},\;0.25,\;0.5,\;0.75,\;1+1\times 10^{-6},\;1.25,\;1.5,\;1.75,\;2\right\}$—from the Equation (6);
- Weights ∈{0.001, 0.005, 0.01, 0.05, 0.1}, which is the hyper-parameter associated with the Rényi or semantic regularization term in Equation (2);
- Batch size ∈{20, 50, 100},which is the mini-batch size needed for adaptive stochastic gradient descent optimization algorithm;
- Number of labeled examples ∈{100, 1000, 50,000}, which is the number of randomly chosen labeled examples from the training set with the assumption that the final set is balanced, i.e., no particular class is overrepresented.

It would purely be an explanatory analysis, giving an insight into the investigated phenomena.

### 4.5. Benchmarking Models

In order to compare and assess the quality of our proposed methods, the following benchmark algorithms are used in the experiment. The first one is the AtlasRBF algorithm, which uses manifold-based kernel for semi-supervised and supervised learning [36] and based on this a classifier learns from existing descriptions of manifolds that characterize the manifold as a set of piecewise affine charts, or an atlas. The second algorithm is the deep generative model, which employs rich parametric density estimators formed by the fusion of probabilistic modelling and deep neural networks [37]. The third algorithm is virtual adversarial training, which is a regularization method based on virtual adversarial loss measuring local smoothness of the conditional label distribution for a given input [10]. The fourth algorithm is the ladder net model, which is trained to simultaneously minimize the sum of supervised and unsupervised cost functions by backpropagation, avoiding the need for layer-wise pretraining [35]. The fifth algorithm is ResNet, where the authors explicitly reformulate the layers as learning residual functions with reference to the layer inputs, instead of learning unreferenced functions [38]. Finally, the last baseline is the base model (denoted later as MLP) described in Section 4.4 which is trained without additional regularization term.

## 5. Empirical Analysis

**Figure 6:**
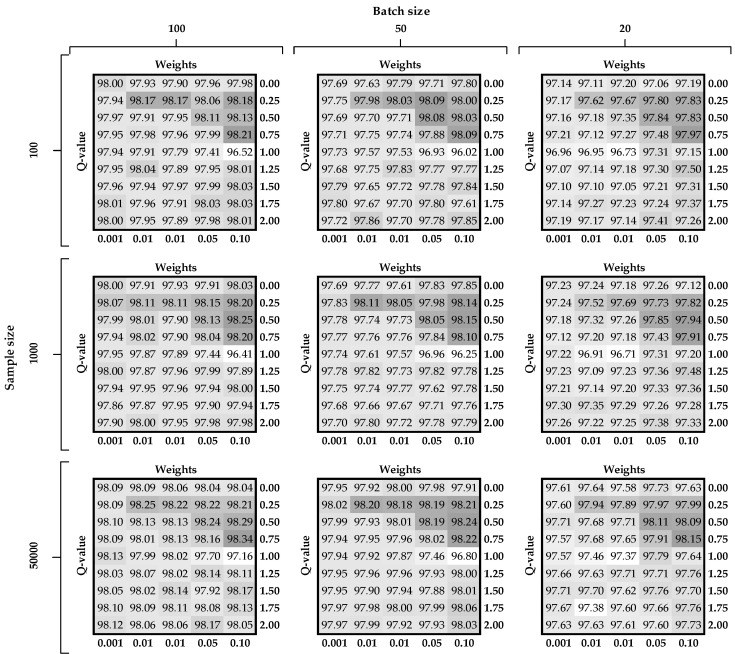
Accuracy on the test set of the MNIST dataset in terms of different combinations of the tuned parameters.

**Figure 7:**
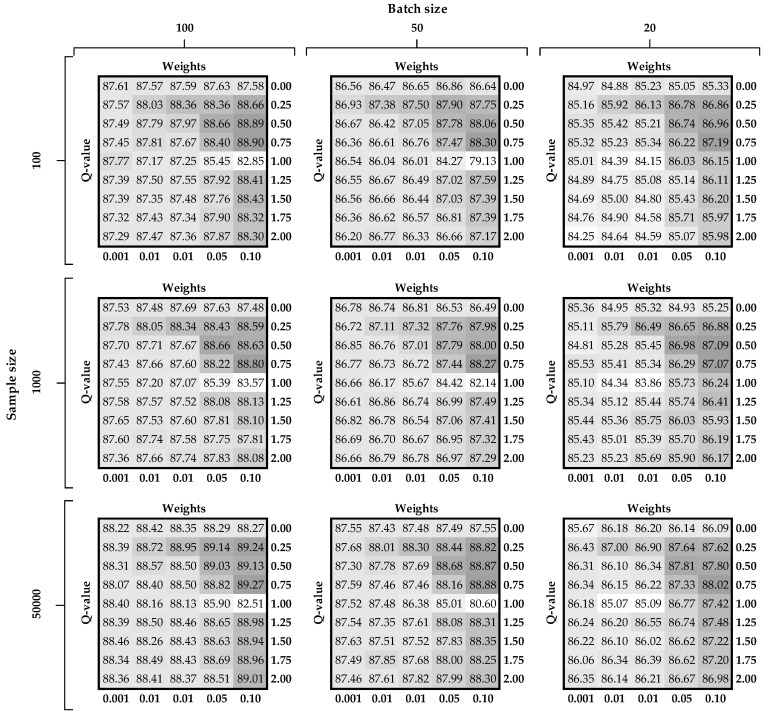
Accuracy on the test set of the Fashion-MNIST dataset in terms of different combinations of the tuned parameters.

In this section, we describe several experiments to test and to compare the performance (using 10-fold cross-validation) of DNNs with regard to semantic loss and generalized entropy loss functions in terms of different settings of the tuned parameters. The comparisons are depicted in Figure 6 and Figure 7, for MNIST and Fashion-MNIST datasets, respectively, and these are prepared in the following manner. On the left-hand side of each figure there are three buckles that denote the number of labeled examples used for the supervised part of the learning.

At the top of each chart there are three buckles pointing to the batch size used for stochastic gradient descent optimization algorithm. There are nine different tables (subfigures) inside the figure for each combination of the aforementioned parameters. Each table on the horizontal axes has the hyper-parameter value related to the regularization term (labels are at the top and values at the bottom of the subfigure). On the vertical axes, there are different values of the Q-parameter used in the Rényi loss function (labels are on the left and values are on the right of the subfigure). At the intersection of the last two described parameters there are accuracy measures (Equation (10)) calculated for the testing sample. Finally, we introduce a color palette for easier identification of promising intersections. Dark gray denotes situations when accuracy is the highest while white indicates areas with the lowest accuracy.

First, we consider Figure 6 with the results for the basic MNIST dataset. In terms of the hyper-parameter for the regularization term, it can be seen that the worst results are obtained when w equals 0.001. In general, while increasing this parameter, the accuracy is gradually improving, achieving the best results when w is set to 0.1. This observation clearly supports our assumption that including Rényi loss as a regularization term for semi-supervised task results in improved classification accuracy. By comparing the w-parameter with the Q-parameter, one can distinguish an upper triangular matrix (dark-gray color) with the highest possible accuracy results. This is observed in all nine subfigures for w ranging between 0.0001 and 0.1 and Q ranging between 0.25 and 0.75. In terms of sample and batch size, as one would expect, accuracy increases when both parameters increase. This means that the average accuracy for 100 labeled training examples with a batch size of 20 is just over 97 (right upper subchart), while that of 50,000 labeled examples with a batch size of 100 is above 98.

Accuracy results for the Fashion-MNIST dataset present very similar characteristics as for the basic MNIST dataset (please see Figure 7). However, there are two main differences. First, the accuracy results oscillate around 83 and 89, which means that this dataset is not yet sufficiently worked out. The second difference is that the results are more diverse with a larger range of variance.

In order to compare results for the semantic loss and the Rényi loss, Table 2 and Table 3 are prepared (together with results for other benchmark models described in Section 4.4). Both tables report the best accuracy results obtained by the following procedure: (1) We derive accuracy for all combinations of the tuning parameter for both losses, i.e., four parameters for the Rényi loss and three parameters for the semantic loss (without Q-parameter) and (2) since these results are computed for training, validation, and test sets, we chose the parameter combination having the best results for the validation sets to report the best mean accuracy for the testing sets (sixth column for both tables). Both tables report the mean accuracy obtained after 10-fold cross-validation together with the standard deviation error of the estimates in the brackets.

As presented in Table 2, when labeled sample size is 100, the best results for both datasets are obtained using weight w set at 0.005. In other situations, w is tuned to 0.1 for both losses. Since using more samples in gradient computation usually gives better estimation, this parameter is tuned to 100 observations for all cases. According to the observations obtained after analyzing Figure 6 and Figure 7 (regarding upper triangular matrix), the best results are associated with the Q-parameter set at 0.5 or 0.75.

In line with the highlighted results in both tables (bolded values), we conclude that the Rényi loss outperforms results related to the semantic loss (there is only one exception for the validation set for the Fashion-MNIST dataset). The biggest difference for basic MNIST is observed for labeled sample size equaling 100 and 1000, i.e., difference is 0.23. For the Fashion-MNIST dataset the biggest difference is noted for the 100 labeled sample size (difference is 1.24).

Moreover, both tables compare both losses (i.e., Rényi and semantic loss) to a baseline MLP and the state-of-the-art results from the literature. The baseline, in this case, is a purely supervised multilayer perceptron, which makes no use of unlabeled data [6,7]. For example, given 100 labeled examples, the DNN with semantic loss gains around 25% (MNIST) and 26% (Fashion-MNIST) improvement over the purely supervised baseline. Finally, the DNN with Rényi loss function gains around 25% (MNIST) and 28% (Fashion-MNIST). Considering the only change is an additional loss term, these results are encouraging.

## 6. Conclusions

Semi-supervised learning is often considered to be a key challenge for future deep learning tasks. We have demonstrated that both semantic loss and Rényi loss provide significant benefits in semi-supervised classification. Application of both losses to the feedforward neural network architecture using unlabeled observations increase the predictive power of the classifiers. The main advantage of both losses is that they only require a simple additional loss term. Without changing the architecture of the DNN itself, it incurs almost no computational overhead. Conversely, this property makes the proposed methods sensitive to the underlying model’s performance. Without the basic predictive power of a strong supervised learning model, it is not expected to see the same benefits that has been seen in this article.

With our analysis, we confirm that improving classification accuracy in semi-supervised classification tasks using semantic loss function and generalized entropy loss is feasible and can be achieved with reasonable accuracy as compared with the base models (first research question). This statement is supported by the results presented in Table 2 and Table 3. Interestingly, applying Rényi loss provides classification improvement up to 28%. Our answers to the second research question regarding the relation between semantic loss and generalized entropy loss reveal that the semantic loss is less sensitive to the class distribution than the error measures (Shannon entropy and Gini index), but at the same time is more sensitive than the miss/classification error. Additionally, as the Q-parameter in Rényi loss increases, sensitivity to the class distribution decreases. After quantifying the relation between these two losses, it turns out that the relationship is functional and can be approximated by a fifth degree polynomial. We have also empirically confirmed that Rényi loss is bounded by the semantic loss. Finally, we have showed that proper tuning of the input parameters improves the final results. By intersecting the tuned parameters, we distinguish an upper triangular matrix with the highest possible accuracy results. 

Finally, we have chosen to investigate semantic loss and Rényi entropy in the same paper because of the following reasons: Semantic loss has its roots in knowledge representation, while Rényi entropy is more familiar to researchers doing statistical machine learning. In addition to functioning as a regularization term, on the one hand, semantic loss is not invented to be a regularization term. On the other hand, researchers almost always view Rényi entropy as a regularization term in the context of learning. It is a fascinating fact that those two seemingly orthogonal things have very similar effects as loss functions, achieving similar results in this setting.

In future work, we plan to investigate whether applying semantic and Rényi losses on different DNN architectures would yield an even stronger performance improvement.

## Figures and Tables

**Table 1:** A k×k confusion matrix for k-class classification problem.

		Predicted Value			
		Class 1	Class 2	⋯	Class *k*
**Real value**	**Class 1**	True_1_	False_1_	⋯	False_1_
	**Class 2**	False_2_	True_2_	⋯	False_2_
	⋮	⋮	⋮	⋱	⋮
	**Class k**	False*_k_*	False*_k_*	⋯	True*_k_*

**Table 2:** Comparison of the best results for Rényi and semantic losses, along with benchmark models on the MNIST dataset.

Sample Size	Loss	Q-Value	Weight	Batch Size	Mean Validation Accuracy	Mean Test Accuracy
100	Semantic		0.005	100	98.02 (∓0.04)	97.98 (∓0.04)
	Rényi	0.75	0.100	100	**98.20** (∓0.02)	98.21 (∓0.03)
	MLP			100		78.46 (∓1.94)
	AtlasRBF					91.9 (∓0.95)
	Deep Generative					96.67 (∓0.14)
	Virtual Adversarial					97.67
	Ladder Net					**98.94 (∓0.37)**
1000	Semantic		0.100	100	97.95 (∓0.05)	98.02 (∓0.03)
	Rényi	0.50	0.100	100	**98.27** (∓0.03)	98.25 (∓0.03)
	MLP			100		94.26 (∓0.31)
	AtlasRBF					96.32 (∓0.12)
	Deep Generative					97.60 (∓0.02)
	Virtual Adversarial					98.64
	Ladder Net					**99.16 (∓0.08)**
50,000	Semantic		0.100	100	98.13 (∓0.03)	98.15 (∓0.04)
	Rényi	0.50	0.100	100	**98.29** (∓0.02)	98.29 (∓0.03)
	MLP			100		98.13 (∓0.04)
	AtlasRBF					98.69
	Deep Generative					99.04
	Virtual Adversarial					99.36
	Ladder Net					**99.43 (∓0.02)**
	ResNet					99.40

**Table 3:** Comparison of the best results for Rényi and semantic losses, along with benchmark models on the Fashion-MNIST dataset.

Sample Size	Loss	Q-Value	Weight	Batch Size	Mean Validation Accuracy	Mean Test Accuracy
100	Semantic		0.005	100	**88.65** (∓0.11)	87.65 (∓0.07)
	Rényi	0.50	0.100	100	89.62 (∓0.07)	**88.89** (∓0.10)
	MLP			100		69.45 (∓2.03)
	Ladder Net					81.46 (∓0.64)
1000	Semantic		0.100	100	88.71 (∓0.06)	87.83 (∓0.07)
	Rényi	0.75	0.100	100	**89.54** (∓0.35)	**88.80** (∓0.08)
	MLP			100		78.12 (∓1.41)
	Ladder Net					86.48 (∓0.15)
50,000	Semantic		0.100	100	89.26 (∓0.08)	88.49 (∓0.10)
	Rényi	0.50	0.100	100	**89.90** (∓0.06)	89.03 (∓0.06)
	MLP			100		88.26 (∓0.18)
	Ladder Net					90.46
	ResNet					**92.00**
